# Oral Cancer Awareness, Screening Behavior, and Risk-Factor Recognition Among Patients Attending a University Dental Hospital

**DOI:** 10.3390/healthcare14152393

**Published:** 2026-08-04

**Authors:** Elif Betul Yıldırım, Ozgun Yıldırım, Yeliz Kılınç, Nur Mollaoğlu

**Affiliations:** 1 Department of Oral and Maxillofacial Surgery, Faculty of Dentistry, Gazi University, Ankara 06490, Turkey; ykilinc@gazi.edu.tr (Y.K.); nurmolla@gazi.edu.tr (N.M.); 2 Department of Oral and Maxillofacial Surgery, Gülhane Faculty of Dentistry, Health Sciences University, Ankara 06490, Turkey; ozgunyldrm89@gmail.com

**Keywords:** early detection, oral cancer, public awareness, questionnaire survey, cross-sectional study, Türkiye

## Abstract

**Background:** Oral cancer remains a major global health concern, with increasing incidence and mortality, especially in developing countries. Public awareness is crucial for early detection and improved outcomes. **Objective:** To assess oral cancer awareness, recognition of risk factors and early signs, screening behavior, and care-seeking preferences among patients attending a university dental hospital in Türkiye. **Methods:** This cross-sectional, facility-based survey included 1360 volunteer patients who attended the Faculty of Dentistry between January and December 2025. Consecutive sampling was used to include all eligible and consenting individuals. A structured questionnaire assessed participants’ sociodemographic characteristics, oral cancer awareness, knowledge of symptoms and risk factors, screening history, and lifestyle habits. Data were analyzed using IBM SPSS Statistics 26.0 (IBM Corp., Armonk, NY, USA). Descriptive statistics, chi-square tests, and multivariable logistic regression analyses were performed. Statistical significance was set at *p* < 0.05. **Main outcomes and measures:** The primary outcome was oral cancer awareness (measured by the question “Have you ever heard of oral cancer?”). Secondary outcomes included knowledge of risk factors and early clinical signs, screening history, preferred healthcare contact for suspected oral cancer, and lifestyle factors (tobacco and alcohol). Associations between sociodemographic variables and oral cancer awareness were also evaluated. **Results:** The sample consisted of 1360 participants (56.4% female; mean age: 33.44 ± 14.60 years; range: 18–70). Of the 1377 questionnaires collected, 1360 were complete and included in the final analysis (questionnaire completion rate: 98.8%). Breast cancer was the most recognized cancer (96.9%), whereas only 50.8% of participants had heard of oral cancer. Dentists (39.4%) were the primary source of information. Tobacco use (92.2%) and alcohol consumption (80.3%) were the most frequently identified risk factors, whereas only 58.3% recognized human papillomavirus as a risk factor for oral cancer. In univariate analyses, oral cancer awareness was significantly associated with age, marital status, education level, monthly income, occupation, alcohol use, and the timing of the most recent dental visit (*p* < 0.05). In multivariable logistic regression, undergraduate education (OR = 2.008, 95% CI: 1.135–3.553), higher monthly income (>100,000 TRY vs. <25,000 TRY, OR = 2.398, 95% CI: 1.571–3.658), alcohol use (OR = 1.521, 95% CI: 1.082–2.139), and a dental visit within the previous year (OR = 4.130, 95% CI: 1.590–10.730) remained independently associated with oral cancer awareness. **Conclusions:** Oral cancer awareness in this study population was limited. The findings suggest that targeted educational strategies may be beneficial for improving oral cancer awareness. Strengthening dentists’ preventive role and promoting education through social media may help improve oral cancer awareness and facilitate earlier detection.

## 1. Introduction

Oral cancer (OC) remains a significant global public health challenge. According to the latest GLOBOCAN 2024 estimates, approximately 452,205 new cases and 194,108 deaths occurred worldwide in 2024, ranking it as the 14th most commonly diagnosed cancer and the 15th leading cause of cancer-related mortality [1]. The economic burden of OC is also substantial; for example, the annual cost associated with OC in India is estimated to be approximately 386 million US dollars [2,3]. Excluding skin and thyroid cancers, OC is the second most common head and neck malignancy in Türkiye [4]. OC is prevalent not only in developing countries but also in developed nations, such as the United Kingdom, where more than 50% of patients are diagnosed at advanced stages [2]. These findings emphasize the importance of public education and outcomes research to promote early detection, improve patient outcomes, and reduce treatment costs. OC has a multifactorial etiology. Tobacco and alcohol consumption are the major risk factors; genetic susceptibility, socioeconomic inequalities, and geographic variation also contribute to its development [5,6]. Furthermore, with changes in sexual behavior worldwide, human papillomavirus (HPV) (HPV-16 and HPV-18)-related oropharynx cancers show a rising incidence [7].

Public awareness plays a critical role in the early detection of OC, as knowledge of its risk factors and early signs may encourage timely healthcare-seeking behavior and facilitate earlier diagnosis [8]. Nevertheless, studies from various countries have consistently reported insufficient public knowledge regarding OC, particularly its established risk factors and early clinical manifestations [5,9,10,11,12,13,14,15]. This limited public awareness is particularly concerning because many cases of OC can be identified during routine visual examination of the oral cavity before the disease reaches an advanced stage [6,16].

Despite advances in surgical management, radiotherapy, and chemotherapy, improvements in treatment have not been accompanied by comparable improvements in early diagnosis or public awareness [6,16,17]. Consequently, many patients continue to present with advanced-stage disease, particularly in settings where awareness of OC remains limited [6,12,17,18]. Similarly, recent studies conducted in different countries continue to report insufficient knowledge of OC, particularly regarding its risk factors and early clinical manifestations [1,9,14,15,19].

In Türkiye, the growing burden of OC has become an increasingly important public health concern. Comprehensive research is essential to support public education, promote early detection, expand screening initiatives, and improve community awareness. Previous studies conducted in Türkiye have consistently reported insufficient public awareness of OC, particularly regarding its early signs and established risk factors. Although tobacco and alcohol are widely recognized as major risk factors, awareness of other well-established risk factors and early clinical manifestations remains limited. Most Turkish studies were conducted more than a decade ago and primarily focused on awareness alone. Consequently, contemporary data on screening behavior, care-seeking preferences, and recognition of established risk factors and early clinical signs among Turkish dental patients remain limited. Therefore, the present study aimed to provide an updated assessment of OC awareness, recognition of risk factors and early signs, screening behavior, and care-seeking preferences among dental patients in Türkiye.

This study aimed to evaluate the level of awareness regarding OC among patients attending the Faculty of Dentistry at Gazi University. The study hypothesized that participants would have limited knowledge of OC risk factors, early clinical signs, and screening behaviors, and that OC awareness would be associated with sociodemographic characteristics, including age, education, income, occupation, and dental attendance. The specific aims of the study were (1) to assess participants’ knowledge of OC risk factors and early clinical signs, (2) to evaluate their awareness of and participation in screening practices, and (3) to identify sociodemographic characteristics associated with differences in awareness and lifestyle habits.

## 2. Methods

### 2.1. Study Design and Setting

This was a cross-sectional, facility-based study conducted in accordance with the STROBE (Strengthening the Reporting of Observational Studies in Epidemiology) guidelines to ensure transparency and reproducibility.

The research protocol was approved by the Clinical Research Ethics Committee of the Faculty of Dentistry, Gazi University (Document Date and Number: 30.01.2020-E.3408).

The study was conducted using face-to-face questionnaires administered to 1360 volunteer patients who presented to the Department of Oral and Maxillofacial Surgery at the Gazi University Faculty of Dentistry in Ankara, Türkiye, between 1 January and 31 December 2025.

### 2.2. Study Population

Data were collected anonymously to ensure confidentiality, and no prior information about OC was provided to participants. Informed consent was obtained from all participants, and the study was conducted in accordance with the principles of the Declaration of Helsinki.

**Inclusion criteria:**

- Patients aged 18 years or older.
- Patients attending the dental clinic for various dental reasons during the study period.
- Individuals with no previous diagnosis of OC.
- Individuals who voluntarily agreed to participate and provided informed consent.

**Exclusion criteria:**

- Individuals with a previous diagnosis of OC.
- Healthcare professionals (physicians, dentists, nurses, or medical students).
- Individuals who were unable to provide informed consent
- Questionnaires were excluded from the final analyses if informed consent was not provided or if more than two questionnaire items (approximately 20% of the questionnaire) were unanswered. The preliminary questionnaire was independently reviewed by two experienced oral and maxillofacial surgeons (Y.K. and N.M.) to assess its face and content validity. Minor revisions were made based on their recommendations. Subsequently, the questionnaire was pilot-tested in 20 adult dental patients to evaluate the clarity, comprehensibility, wording, feasibility of administration, and completion time. Minor wording revisions were made based on participant feedback, and the pilot participants were excluded from the final analysis.

### 2.3. Sampling and Sample Size Calculation

A non-probability consecutive sampling method was used. All eligible patients who attended the Department of Oral and Maxillofacial Surgery during the study period, between 1 January and 31 December 2025, were invited to participate consecutively.

The minimum sample size required to estimate a single population proportion was calculated using the following formula:

n = Z^2^p(1 − p)/d^2^

A 95% confidence level (Z = 1.96), an expected proportion of 0.50, and an absolute margin of error of 0.03 were used. The expected proportion was set at 0.50 because it provides the maximum required sample size when the population proportion is unknown.

n = (1.96^2^ × 0.50 × 0.50)/0.03^2^ = 1067.11

Minimum required sample size = 1068 participants.

A total of 1377 questionnaires were collected, exceeding the minimum required sample size of 1068 participants. Because the total number of eligible patients invited to participate was not prospectively recorded, an exact participation rate could not be calculated. Of the 1377 questionnaires collected, 17 were excluded due to substantial missing data, yielding a final analytical sample of 1360 participants (questionnaire completion rate: 98.8%).

### 2.4. Data Collection

During the study’s planning phase, a comprehensive literature review was conducted to identify previously published studies and questionnaires assessing OC awareness, knowledge, and recognition of risk factors [15,20,21,22,23,24]. Relevant articles were identified through searches of PubMed, Scopus, and Google Scholar using combinations of the keywords “OC awareness,” “OC knowledge,” “OC risk factors,” “oral health,” and “questionnaire.” The questionnaire was developed specifically for the present study and was not a direct translation or adaptation of a single previously validated instrument. Instead, relevant items were selected and adapted from previously published OC awareness questionnaires. Minor modifications were made to improve clarity, cultural appropriateness, and consistency with the objectives of the present study and the Turkish context [23,24].

The final questionnaire consisted of 12 structured questions with dichotomous, multiple-choice, and multiple-response formats. Knowledge-related items included the response options “Yes,” “No,” and “I do not know.” No composite knowledge or awareness score was calculated; each item was analyzed separately. The questionnaire consisted exclusively of structured questions with predefined response options and did not include any open-ended questions. Therefore, no qualitative data were collected.

Data were collected through face-to-face administration of the questionnaire in the clinic waiting area, ensuring that participants relied solely on their own knowledge and did not consult online information. Each session lasted approximately 15 to 20 min. To minimize the possibility of duplicate responses, the questionnaire was administered only to patients attending the Faculty of Dentistry for their initial visit during the study period. As only first-time attendees were eligible, each participant could be enrolled only once.

When participants had difficulty reading or writing, the oral and maxillofacial surgery specialist administering the questionnaires assisted them. Participants who answered “No” to the question “Have you ever heard of OC?” were instructed to skip Questions 3–8 and proceed directly to Question 9. Consequently, only participants who reported prior awareness of OC completed the subsequent awareness-related questions.

The final questionnaire covered the following seven domains:

1. Socio-demographic features;
2. Knowledge and awareness of OC;
3. Source of information about OC;
4. Knowledge of clinical signs and symptoms of OC;
5. Knowledge of risk factors for OC;
6. Whether participants had ever screened for OC, which type of healthcare professional they would consult first in case of suspected OC, and the timing of their most recent dental visit;
7. Lifestyle habits, including tobacco and alcohol use and their frequency.

All questionnaires were administered by a specialist in oral and maxillofacial surgery with over 6 years of clinical experience and trained in standardized communication and data collection procedures.

### 2.5. Outcome Measures

The primary outcome of the study was awareness of OC, determined by participants’ response to the question “Have you ever heard of OC?” (Yes/No). This variable represented the general level of recognition of OC within the study population. No composite OC knowledge or awareness score was calculated. General awareness was assessed using the question “Have you ever heard of OC?”, whereas knowledge of OC signs, symptoms, and risk factors was evaluated separately for each questionnaire item. Therefore, participants were not categorized into low-, moderate-, or high-knowledge groups.

The secondary outcomes included several domains assessing participants’ understanding, experiences, and behaviors related to OC. These comprised knowledge of major risk factors (such as tobacco use, alcohol consumption, HPV infection, and chronic irritation), and knowledge of early clinical signs and symptoms, including non-healing oral ulcers, red or white patches, and persistent oral pain. In addition, participants were asked about their sources of information regarding OC (e.g., dentists, or traditional media (television and radio), social media), as well as their screening experiences and healthcare-seeking behaviors, including whether they had ever undergone OC screening, which type of healthcare professional they would first consult in the case of a suspected lesion, and the timing of their most recent dental visit. Participants’ lifestyle habits, including the use and frequency of use of tobacco and alcohol, were also recorded to explore their possible association with awareness and knowledge levels.

Furthermore, sociodemographic characteristics such as age, sex, education level, occupation, place of residence, and income status were collected and treated as covariates to evaluate their potential influence on OC awareness, knowledge of risk factors, and preventive behaviors.

### 2.6. Statistical Analysis

Statistical analyses were performed using IBM SPSS Statistics for Windows, Version 26.0 (IBM Corp., Armonk, NY, USA). Descriptive statistics were used to summarize the dataset’s general characteristics. Categorical variables are presented as frequencies and percentages [*n* (%)], whereas continuous variables are summarized as means and standard deviations (mean ± SD). The primary outcome variable was OC awareness (“Have you ever heard of OC?”). Bivariate associations between OC awareness and sociodemographic and behavioral variables (sex, age, marital status, education level, occupation, monthly income, place of residence, alcohol use, tobacco use, and timing of the most recent dental visit) were examined using the chi-square (χ^2^) test. Cramér’s V (or phi coefficient for 2 × 2 tables) was used to quantify the strength of the associations, with values of 0.10, 0.30, and 0.50 interpreted as small, medium, and large effect sizes, respectively. Variables showing statistically significant associations (*p* < 0.05) in the bivariate analyses were entered into binary logistic regression models. Crude associations were first evaluated using separate univariate logistic regression models, after which all candidate variables (age, marital status, education level, monthly income, occupation, alcohol use, and timing of the most recent dental visit) were entered simultaneously into a multivariable model using the forced-entry method. All categorical predictors were entered using indicator coding with prespecified reference categories.

No formal adjustment for multiple comparisons was applied because the bivariate analyses were exploratory and intended to identify potential factors associated with OC awareness. Accordingly, *p*-values were interpreted together with effect size measures and the results of the multivariable logistic regression analysis.

The strength of associations was expressed as odds ratios (ORs) with 95% confidence intervals (CIs), and a two-sided *p*-value < 0.05 was considered statistically significant. The overall performance of the multivariable logistic regression model was evaluated using the omnibus likelihood-ratio test, −2 log-likelihood, Cox and Snell R^2^, Nagelkerke R^2^, and the Hosmer–Lemeshow goodness-of-fit test. Classification performance was assessed using overall classification accuracy, sensitivity, and specificity. Multicollinearity was evaluated using tolerance and variance inflation factor (VIF) values, whereas potentially influential observations were assessed using standardized residuals, Cook’s distance, and leverage values.

**Figure 1:**
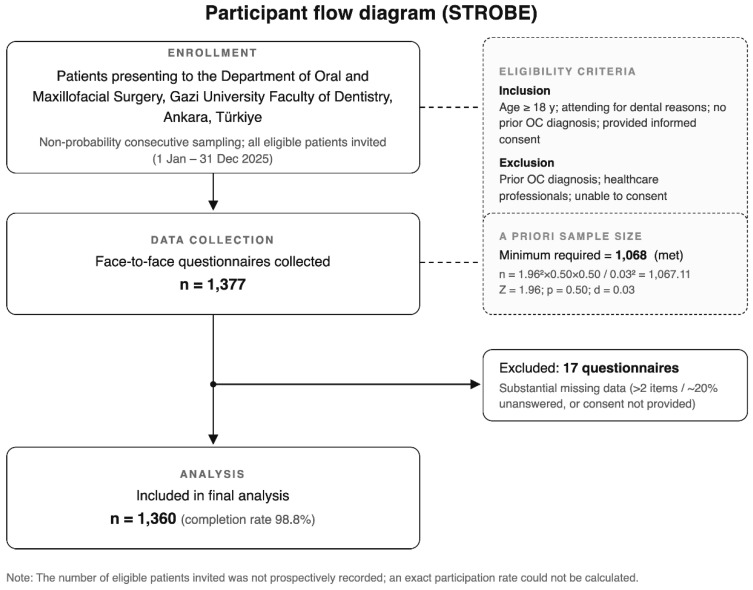
Flow diagram illustrating participant recruitment, eligibility assessment, exclusions, and inclusion in the final analysis according to the STROBE recommendations.

Percentages were calculated using the number of valid responses for each variable. Therefore, the denominators vary across variables because of missing data. The participant recruitment and selection process is summarized in Figure 1.

## 3. Results

A total of 1360 participants were included in the study. The sociodemographic characteristics of the participants are presented in Table 1.

### 3.1. Awareness of Different Cancer Types

Among the cancer types assessed, breast cancer was the most widely recognized (96.9%, *n* = 1241), followed by lung cancer (94.9%, *n* = 1216) and leukemia (90.8%, *n* = 1163). Colorectal cancer and uterine cancer were recognized by 85.8% of participants. In this multiple-response question, head and neck cancer had the lowest level of awareness (46.4%; Table 2).

### 3.2. Sources of Information About OC

Among participants who were aware of OC, dentists were the most frequently reported source of information (39.4%), followed by social media/internet and traditional (TV and radio) media (Table 2).

### 3.3. Awareness of the Early Signs of OC

Non-healing oral ulcers were the most frequently recognized sign of OC (83.8%). Awareness of other clinical signs was considerably lower, particularly for ill-fitting dentures and voice changes (Table 2).

### 3.4. Awareness of the Risk Factors Associated with OC

Tobacco use was the most frequently identified risk factor (92.2%), followed by alcohol consumption (80.3%). In contrast, awareness of HPV-related risk remained comparatively limited (58.3%) (Table 2).

### 3.5. Awareness of OC Prevention and Treatment

Only 5.1% of participants reported having undergone OC screening. Most participants indicated that they would first consult a physician rather than a dentist if they noticed a suspicious oral lesion (Table 2).

### 3.6. OC Awareness and Associated Factors

OC awareness was significantly associated with age, marital status, education level, monthly income, occupation, alcohol consumption, and the timing of the most recent dental visit (all *p* < 0.05; Table 3). No significant associations were observed with sex, place of residence, or tobacco use (all *p* > 0.05). Awareness was generally higher among younger participants, unmarried individuals, those with higher educational attainment and income, students, alcohol users, and participants who had visited a dentist more recently.

### 3.7. Logistic Regression Analysis of Factors Associated with OC Awareness

The multivariable logistic regression model was statistically significant and demonstrated acceptable model fit (Nagelkerke *R*^2^ = 0.162; Hosmer–Lemeshow *p* = 0.076). In univariate analyses, age, marital status, education level, monthly income, occupation, alcohol use, and the timing of the most recent dental visit were significantly associated with OC awareness (Table 4). In the multivariable model, education level, monthly income, alcohol use, and recent dental attendance remained independently associated with OC awareness, whereas age, marital status, and occupation were no longer significant after adjustment (Table 4).

### 3.8. The Impact of Alcohol Consumption on Sociodemographic Characteristics

Alcohol consumption was significantly associated with sex, age, occupation, marital status, education level, place of residence, and monthly income (all *p* < 0.05; Table 5). Alcohol use was generally more common among men, unmarried participants, individuals with higher education and income, and those in younger age groups.

### 3.9. The Impact of Tobacco Use on Sociodemographic Characteristics

Tobacco use was significantly associated with sex, age, occupation, education level, and monthly income (all *p* < 0.05), but not with marital status or place of residence (Table 6). Tobacco use was more common among men, middle-aged participants, self-employed individuals, and those with higher monthly incomes.

## 4. Discussion

This study provides an updated assessment of OC awareness among dental patients in Türkiye. Although participants demonstrated relatively good recognition of tobacco use and non-healing oral ulcers as risk factors and early signs, awareness of other established risk factors, particularly HPV infection and sun exposure, remained limited. In addition, participation in OC screening was extremely low, highlighting important gaps in public awareness and preventive practices. These findings suggest that awareness is selective rather than comprehensive and emphasize the need for broader public education strategies that extend beyond traditional tobacco-related messages.

Data regarding the epidemiological profile of OC in Türkiye remain limited [4]. Anticipating a substantial increase in the overall cancer burden—particularly in developing countries—the Turkish Ministry of Health published a report on national cancer statistics in 2025. According to this report, cancer accounted for one in every six deaths in Türkiye in 2022 [25]. Moreover, projections indicate that OC cases are expected to increase significantly in the coming years [1,26]. These trends underscore the need to develop effective prevention strategies and implement comprehensive early detection protocols to mitigate the growing burden of OC [27].

The World Health Organization (WHO) also projects a continued global increase in OC cases over the coming decades [28]. Tobacco use and alcohol consumption are considered the most significant contributors to OC [15]. In Western countries, tobacco use is regarded as one of the most critical risk factors for oral OC, with the tongue being the most commonly affected site. Chewing tobacco and betel areca nut predominantly affect the buccal mucosa in Asian countries [3]. OC is strongly associated with lifestyle-related risk factors and individual habits, and numerous studies have demonstrated that men are more likely to engage in high-risk behaviors such as tobacco and alcohol consumption, particularly as they age [24,26,29]. According to the present study, a higher prevalence of tobacco and alcohol consumption was observed among male patients. These demographic findings are broadly consistent with previous reports [17,29].

Early detection of suspicious lesions significantly improves the five-year survival rate associated with stage I and II OCs. In contrast, survival outcomes decrease markedly in advanced-stage lesions (stages III and IV) [5]. Moreover, early diagnosis may also reduce the economic burden of OC, as advanced-stage disease requires substantially more extensive and costly treatment than early-stage disease [29]. While increasing awareness of OC among dental professionals is essential, it is equally important for patients to be aware of the need to consult a healthcare provider when they notice any changes in the oral cavity [27,28]. Alcohol use also remained independently associated with greater OC awareness. This finding should not be interpreted as alcohol having a protective effect; rather, it may reflect differences in education, lifestyle, exposure to health information, or awareness of alcohol-related cancer risks. Overall, the findings indicate that public awareness of OC remains incomplete, particularly regarding its clinical manifestations and several established risk factors [5,16,30].

Compared with the earlier Turkish study by Peker and Alkurt, the present study demonstrated higher awareness of oral cancer and better recognition of major risk factors. However, participation in oral cancer screening remained low, suggesting that increased awareness has not yet translated into preventive behaviors. The observed improvement in awareness may reflect increased public access to health information, wider use of digital media, greater emphasis on preventive healthcare, and enhanced patient education during dental visits. Nevertheless, these comparisons should be interpreted with caution because differences in study populations, sampling strategies, questionnaire design, and the time periods during which the surveys were conducted may also have contributed to the observed differences [23]. Hafed et al. and Rai et al. also reported significantly higher levels of OC awareness among younger individuals, consistent with our univariate findings. However, age was no longer independently associated with OC awareness after adjustment for other sociodemographic variables in the multivariable analysis [9,14]. Nevertheless, age-related differences in awareness may vary depending on the characteristics of the study population and the sociocultural context in which the study was conducted [31]. Similarly, our study showed that awareness was higher among participants with greater educational attainment, while important gaps persisted in OC screening and recognition of some established risk factors [23,31,32]. Similar findings have also been reported in studies from Malaysia, Palestine, Singapore, Germany, and several Middle Eastern and North African countries, where awareness of tobacco as a major risk factor was relatively high, whereas knowledge of HPV infection, alcohol consumption, and other established risk factors, as well as OC screening, remained limited [12,13,14,19,33].

Pictorial health warnings on cigarette packs are well-designed and cost-effective global tobacco control strategies, given their broad population-level access [34]. WHO encourages the implementation of graphic health warnings on tobacco products as part of its comprehensive approach to reducing the number of smokers worldwide. As of October 2020, 17 countries, including Türkiye, had officially adopted this policy for various tobacco products [35]. Although 92.2% of participants in this study identified tobacco use as a risk factor for OC, 34.4% still reported current tobacco consumption. This finding is consistent with previous research suggesting that awareness of the adverse health consequences of tobacco use does not necessarily lead to behavioral change or reduced usage. These results underscore the importance of combining pictorial health warnings with broader behavioral interventions and public education efforts to achieve more effective tobacco control [5,16,36]. Many of these studies have reported that individuals who smoke demonstrate a higher level of awareness of OC compared to non-smokers [5]. These findings highlight the need for implementing extensive oral health education programs, motivational questioning, group therapies, and counseling interventions [5,36].

OC mortality rates have not demonstrated a significant decrease, even though there have been advancements in surgical techniques, reconstructive procedures, radiotherapy, and chemotherapy [28]. Limited public awareness remains a major barrier to early detection and improved survival outcomes [37]. A study was also conducted in Afghanistan among 435 participants, who reported that only 41.8% were aware of OC; even lower rates were observed in Iran (30.7%) and Nigeria (15.3%) [5]. Several countries, including Portugal, the United States, China, and Saudi Arabia, have reported that the awareness of OC exceeds 50% among their populations [5]. Another investigation focusing on individuals diagnosed with precancerous lesions revealed that only half of the participants knew about OC [36]. These findings highlight the critical necessity for implementing public awareness campaigns, enhanced health literacy, and integrating screening programs into national health strategies to reduce the burden of OC effectively.

In Germany, Baumann et al. developed and implemented a campaign to enhance the early diagnosis of OC through various media strategies targeting individuals over 50, educationally disadvantaged groups, and women [17]. The particular focus on women may be related to sociocultural dynamics, as women typically demonstrate greater awareness and generally more positive attitudes toward health-related information, for themselves and their family members. Similarly, in the present study, women showed higher participation levels than men. Participants who reported knowing OC were asked about their sources of information. The primary sources were dentists, followed by traditional media (television and radio), and social media. In contrast to the present findings, other studies have found that the media is a more prominent source of information. These differences may also reflect variations in healthcare systems, public health campaigns, educational attainment, cultural attitudes toward cancer prevention, and the use of mass media across countries [17,36,38].

In the current study, only 5.1% of participants reported having undergone OC screening. Increasing participation in screening initiatives requires not only improving public awareness but also facilitating access to preventive oral healthcare services [29,39]. Several studies have demonstrated that patients frequently lack understanding of the early signs and symptoms of OC, show low engagement in screening programs, and possess limited knowledge about relevant risk factors [5,16,17,18,40]. Because OC can be detected through routine visual examination of the mouth without complex screening procedures, increasing awareness among both the public and healthcare professionals may substantially improve opportunities for early diagnosis [29,39]. Routine opportunistic oral examinations performed by trained primary healthcare providers have also been identified as a practical and cost-effective strategy for improving early detection, particularly among high-risk populations [41]. Therefore, public health initiatives should promote routine oral examinations, particularly for high-risk individuals, through multidisciplinary educational strategies involving dentists, physicians, nurses, pharmacists, and public health professionals [39]. Community-based educational campaigns and evidence-based educational materials may further enhance awareness and encourage timely referral of suspicious lesions [41].

Beyond improving public awareness, equitable access to healthcare services is important for the timely diagnosis of OC. Individuals without health insurance, those living in rural areas, and ethnic minorities have been reported to have more limited access to dental services. In addition, healthcare professionals’ knowledge and awareness of OC, as well as patients’ comorbidities, may influence the diagnostic and treatment process. Cancer-related health inequities are also influenced by educational level, socioeconomic status, preventable risk factors, and structural barriers within healthcare systems, including limited primary care capacity, delayed referral pathways, and restricted diagnostic services. These barriers may further widen disparities in OC outcomes [41].

Therefore, reducing disparities in OC outcomes requires not only improving public awareness but also implementing long-term public health strategies that promote equitable access to preventive oral healthcare, early diagnosis, and multidisciplinary collaboration among healthcare professionals [42,43]. Strengthening primary healthcare services and integrating opportunistic OC screening into routine dental and primary care visits may represent an effective approach to reducing diagnostic delays and improving access to early detection [41]. Similarly, improving OC awareness requires a multidisciplinary approach involving dentists and other primary healthcare professionals to promote early detection and timely referral [42].

In Türkiye, the Ministry of Health has established Cancer Early Diagnosis, Screening, and Training Centers (KETEM) in all provinces to promote early diagnosis and easy access to cancer screening services. These centers mainly focus on breast, cervical, skin, gastric-colon, and prostate cancers, as well as efforts to address tobacco product addiction. Based on the findings of the present study, integrating OC awareness activities and opportunistic screening into existing KETEM programs may represent a feasible public health strategy to improve awareness, promote earlier diagnosis, and reduce inequalities in access to preventive oral healthcare.

## 5. Limitations

This study has several limitations. First, participants were recruited consecutively from a single university dental hospital in an urban setting, which may have introduced selection bias and limited the generalizability of the findings to the broader Turkish population. Second, the questionnaire relied on self-reported information, making the findings susceptible to recall, response, and social desirability biases. Third, awareness was assessed primarily through participants’ recognition and self-reported knowledge rather than objective measures of comprehension or actual preventive behaviors. In addition, the questionnaire did not directly assess participants’ family history of OC; therefore, the independent contribution of family history to OC awareness could not be evaluated. Finally, because of the cross-sectional study design, causal relationships between OC awareness and the associated factors cannot be established. Future studies should include more representative multicenter populations, incorporate family history as an independent variable, and evaluate behavioral outcomes related to OC awareness.

## 6. Conclusions

This study found that public awareness of OC among patients attending a university dental hospital in Türkiye remains insufficient, with limited recognition of risk factors beyond tobacco use and low participation in screening programs. Although recognition of some early signs was relatively high, important knowledge gaps remain. These findings suggest that educational interventions to improve OC awareness, particularly among individuals with lower levels of awareness and those at higher risk, may be beneficial. Future multicenter and community-based studies should evaluate the effectiveness, feasibility, and cost-effectiveness of multidisciplinary educational interventions and opportunistic screening programs, particularly among socioeconomically disadvantaged and high-risk populations.

## Figures and Tables

**Table 1:** Sociodemographic Characteristics of the Participants.

Variable	Category	*n* (%)
Sex	Female	767 (56.4)
	Male	593 (43.6)
Age, years	Mean ± SD (range)	33.44 ± 14.60 (18–70)
Age group	≤25 years	464 (41.0)
	26–30 years	129 (11.4)
	31–39 years	170 (15.0)
	40–49 years	160 (14.1)
	≥50 years	208 (18.4)
Occupation	Unemployed	274 (21.6)
	Student	472 (37.2)
	Public-sector employee	203 (16.0)
	Self-employed	205 (16.2)
	Retired	108 (8.5)
	Private-sector employee	7 (0.6)
Marital status	Married	594 (45.6)
	Single	709 (54.4)
	Other	0 (0.0)
Education level	Primary or secondary school	188 (14.2)
	High school	325 (24.5)
	Undergraduate degree	719 (54.2)
	Master’s degree	79 (6.0)
	Doctoral degree	16 (1.2)
Place of residence	Village	38 (2.9)
	Town	28 (2.2)
	District	184 (14.2)
	City	1045 (80.7)
Monthly income	<25,000 TRY	262 (21.1)
	25,000–50,000 TRY	199 (16.0)
	50,001–100,000 TRY	220 (17.7)
	>100,000 TRY	559 (45.1)

*Totals may vary across variables because of missing data.*

**Table 2:** (**A**) Awareness of Different Types of Cancer. (**B**) General Awareness of OC. (**C**) Sources of Information About OC. (**D**) Awareness of Signs and Symptoms of OC. (**E**) Awareness of Risk Factors for OC. (**F**) Screening, Healthcare-Seeking and Lifestyle Characteristics.

**(A)**		
**Variable**	**Category**	***n* (%)**
**Breast cancer**	**Yes**	**1241 (96.9)**
	No	40 (3.1)
	Missing/excluded	79 (5.8)
Lung cancer	Yes	1216 (94.9)
	No	65 (5.1)
	Missing/excluded	79 (5.8)
Bowel cancer	Yes	1099 (85.8)
	No	182 (14.2)
	Missing/excluded	79 (5.8)
Head and neck cancer	Yes	595 (46.4)
	No	686 (53.6)
	Missing/excluded	79 (5.8)
**(B)**		
**Variable**	**Category**	***n*** **(%)**
Have you ever heard of OC?	Yes	691 (50.8)
	No	669 (49.2)
**(C)**		
**Variable**	**Category**	***n*** **(%)**
Traditional media—television or radio	Selected	216 (33.1)
	Not selected	436 (66.9)
Social media or the internet	Selected	246 (37.7)
	Not selected	406 (62.3)
Newspapers and magazines	Selected	85 (13.0)
	Not selected	567 (87.0)
Family members or friends	Selected	179 (27.5)
	Not selected	473 (72.5)
Dentist	Selected	257 (39.4)
	Not selected	395 (60.6)
Medical doctor	Selected	128 (19.6)
	Not selected	524 (80.4)
Other healthcare professionals	Selected	61 (9.4)
	Not selected	591 (90.6)
**(D)**		
**Variable**	**Category**	***n*** **(%)**
Non-healing wounds in the mouth	Yes	557 (83.8)
	No	25 (3.8)
	Don’t know	83 (12.5)
Painful white lesions in the mouth	Yes	429 (64.9)
	No	66 (10.0)
	Don’t know	166 (25.1)
Ill-fitting dentures	Yes	169 (25.8)
	No	223 (34.0)
	Don’t know	264 (40.2)
Abscess or swelling in the mouth	Yes	365 (55.6)
	No	113 (17.2)
	Don’t know	178 (27.1)
Painful red lesions in the mouth	Yes	450 (68.6)
	No	42 (6.4)
	Don’t know	164 (25.0)
Bleeding gums	Yes	313 (48.1)
	No	157 (24.1)
	Don’t know	181 (27.8)
Numbness in the mouth	Yes	329 (50.9)
	No	97 (15.0)
	Don’t know	221 (34.2)
Swelling in the neck	Yes	350 (54.0)
	No	80 (12.3)
	Don’t know	218 (33.6)
Numbness in the tongue	Yes	356 (54.9)
	No	73 (11.3)
	Don’t know	219 (33.8)
Difficulty chewing or swallowing	Yes	382 (58.9)
	No	74 (11.4)
	Don’t know	193 (29.7)
Voice changes	Yes	294 (45.4)
	No	102 (15.7)
	Don’t know	252 (38.9)
**(E)**		
**Variable**	**Category**	***n*** **(%)**
Smoking	Yes	606 (92.2)
	No	21 (3.2)
	Don’t know	30 (4.6)
Regular alcohol consumption	Yes	525 (80.3)
	No	51 (7.8)
	Don’t know	78 (11.9)
Poor oral health or poor oral hygiene	Yes	522 (80.1)
	No	41 (6.3)
	Don’t know	89 (13.7)
Recreational drug use	Yes	515 (79.2)
	No	39 (6.0)
	Don’t know	96 (14.8)
Genetic factors or family history of cancer	Yes	520 (79.9)
	No	46 (7.1)
	Don’t know	85 (13.1)
Sexual activity or human papillomavirus infection	Yes	373 (58.3)
	No	85 (13.3)
	Don’t know	182 (28.4)
Sun exposure	Yes	262 (41.3)
	No	150 (23.6)
	Don’t know	223 (35.1)
Chronic irritation	Yes	340 (53.7)
	No	99 (15.6)
	Don’t know	194 (30.6)
Excessively hot beverages	Yes	302 (47.5)
	No	133 (20.9)
	Don’t know	201 (31.6)
Spicy foods	Yes	247 (38.9)
	No	165 (26.0)
	Don’t know	223 (35.1)
A diet low in fruits and vegetables	Yes	268 (42.2)
	No	146 (23.0)
	Don’t know	221 (34.8)
Obesity	Yes	222 (35.0)
	No	156 (24.6)
	Don’t know	257 (40.5)
**(F)**		
**Variable**	**Category**	***n*** **(%)**
Is OC contagious?	Yes	96 (14.8)
	No	553 (85.2)
Have you ever been screened for OC?	Yes	34 (5.1)
	No	631 (94.9)
Who would you first consult in case of suspected OC?	Dentist	217 (33.0)
	Medical doctor	440 (67.0)
When was your most recent dental visit excluding today’s appointment?	Never	32 (2.4)
	Within the past year	898 (68.4)
	1–2 years ago	219 (16.7)
	More than 2 years ago	163 (12.4)
Alcohol consumption	No alcohol consumption	979 (73.9)
	Every day	20 (1.5)
	Two or three times per week	73 (5.5)
	Four or more times per week	24 (1.8)
	Once per month or less	148 (11.2)
	Two to four times per month	80 (6.0)
Use of tobacco products	Yes	455 (34.4)
	No	868 (65.6)

*Totals may vary across variables because of missing data.*

**Table 3:** Associations Between Sociodemographic and Behavioral Factors and Awareness of OC.

Variable	Category	Yes, *n* (%)	No, *n* (%)	χ^2^	*p*-Value	Effect Size
Sex	Female	402 (52.4)	365 (47.6)	1.809	0.179	φ = 0.036
	Male	289 (48.7)	304 (51.3)			
Age group	≤25 years	302 (65.1)	162 (34.9)	61.774	<0.001	V = 0.234
	26–30 years	56 (43.4)	73 (56.6)			
	31–39 years	67 (39.4)	103 (60.6)			
	40–49 years	60 (37.5)	100 (62.5)			
	≥50 years	98 (47.1)	110 (52.9)			
Marital status	Married	240 (40.4)	354 (59.6)	44.502	<0.001	φ = 0.185
	Single	418 (59.0)	291 (41.0)			
Education level	Primary/Secondary school	58 (30.9)	130 (69.1)	108.373	<0.001	V = 0.286
	High school	115 (35.4)	210 (64.6)			
	Undergraduate	454 (63.1)	265 (36.9)			
	Master’s	40 (50.6)	39 (49.4)			
	Doctorate	12 (75.0)	4 (25.0)			
Monthly income	<25,000 TRY	108 (41.2)	154 (58.8)	16.344	0.001	V = 0.115
	25,000–50,000 TRY	106 (53.3)	93 (46.7)			
	50,001–100,000 TRY	117 (53.2)	103 (46.8)			
	>100,000 TRY	314 (56.2)	245 (43.8)			
Occupation	Unemployed	94 (34.3)	180 (65.7)	83.162	<0.001	V = 0.257
	Student	311 (65.9)	161 (34.1)			
	Public-sector employee	116 (57.1)	87 (42.9)			
	Self-employed	84 (41.0)	121 (59.0)			
	Retired	54 (50.0)	54 (50.0)			
Place of residence	Village	18 (47.4)	20 (52.6)	1.733	0.630	V = 0.037
	Town	15 (53.6)	13 (46.4)			
	District	86 (46.7)	98 (53.3)			
	City	539 (51.6)	506 (48.4)			
Alcohol use	Yes	212 (61.4)	133 (38.6)	19.873	<0.001	φ = 0.123
	No	465 (47.5)	514 (52.5)			
Use of tobacco products	Yes	241 (53.0)	214 (47.0)	0.972	0.324	φ = 0.027
	No	435 (50.1)	433 (49.9)			
Last dental visit	Never	8 (25.0)	24 (75.0)	23.013	<0.001	V = 0.132
	Within the past year	494 (55.0)	404 (45.0)			
	1–2 years ago	103 (47.0)	116 (53.0)			
	More than 2 years ago	66 (40.5)	97 (59.5)			

**Table 4:** Binary logistic regression analysis of factors associated with OC awareness.

Variables	Univariate Analysis				Multivariable Analysis			
	B	SE	*p*	OR (95% CI)	B	SE	*p*	OR (95% CI)
**Age (ref: ≥50 years)**			<0.001 *				0.115	
**≤** **25 years**	0.738	0.170	<0.001 *	2.092 (1.501–2.918)	−0.013	0.373	0.972	0.987 (0.475–2.049)
**26–30 years**	−0.150	0.226	0.507	0.861 (0.553–1.340)	−0.646	0.336	0.055	0.524 (0.271–1.013)
**31–39 years**	−0.315	0.210	0.133	0.730 (0.484–1.101)	−0.459	0.285	0.107	0.632 (0.362–1.104)
**40–49 years**	−0.395	0.214	0.065	0.673 (0.442–1.025)	−0.385	0.286	0.179	0.680 (0.388–1.192)
**Marital status (ref: Unmarried [single/other])**			<0.001 *				0.942	
**Married**	−0.751	0.113	<0.001 *	0.472 (0.378–0.589)	−0.017	0.238	0.942	0.983 (0.617–1.567)
**Education level (ref: Primary/Secondary)**			<0.001 *				0.009 *	
**High school**	0.205	0.196	0.296	1.227 (0.836–1.802)	0.075	0.277	0.786	1.078 (0.626–1.856)
**Undergraduate**	1.345	0.176	<0.001 *	3.840 (2.721–5.420)	0.697	0.291	0.017 *	2.008 (1.135–3.553)
**Postgraduate (Master’s/PhD)**	0.997	0.260	<0.001 *	2.711 (1.629–4.509)	0.311	0.384	0.417	1.365 (0.643–2.897)
**Monthly income (TRY. ref: <25,000)**			0.001 *				0.001 *	
**25,000–50,000**	0.486	0.190	0.010 *	1.625 (1.121–2.357)	0.563	0.239	0.019 *	1.755 (1.098–2.806)
**50,001–100,000**	0.482	0.184	0.009 *	1.620 (1.128–2.325)	0.620	0.238	0.009 *	1.859 (1.167–2.964)
**>100,000**	0.603	0.152	<0.001 *	1.828 (1.357–2.460)	0.874	0.216	<0.001 *	2.398 (1.571–3.658)
**Occupation (ref: Unemployed)**			<0.001 *				0.134	
**Student**	1.308	0.160	<0.001 *	3.699 (2.703–5.062)	0.491	0.317	0.121	1.634 (0.878–3.041)
**Public-sector employee**	0.937	0.191	<0.001 *	2.553 (1.757–3.709)	−0.022	0.287	0.939	0.978 (0.558–1.716)
**Self-employed**	0.285	0.191	0.135	1.329 (0.915–1.932)	−0.303	0.265	0.252	0.738 (0.439–1.241)
**Retired**	0.650	0.231	0.005 *	1.915 (1.218–3.010)	0.084	0.351	0.810	1.088 (0.547–2.163)
**Alcohol use (Yes; ref: No)**	0.566	0.128	<0.001 *	1.762 (1.372–2.263)	0.420	0.174	0.016 *	1.521 (1.082–2.139)
**Last dental visit (ref: Never)**			<0.001 *				0.002 *	
**Within the past year**	1.300	0.414	0.002 *	3.668 (1.630–8.253)	1.418	0.487	0.004 *	4.130 (1.590–10.730)
**1–2 years ago**	0.980	0.430	0.023 *	2.664 (1.147–6.189)	0.972	0.511	0.057	2.644 (0.971–7.200)
**More than 2 years ago**	0.714	0.438	0.104	2.041 (0.865–4.819)	0.964	0.528	0.068	2.622 (0.931–7.384)

* *p < 0.05. B: regression coefficient; SE: standard error; OR: odds ratio; CI: confidence interval. Model χ* ^2^ *= 113.322 (df = 19. p < 0.001). Nagelkerke R* ^2^ *= 0.162. Hosmer–Lemeshow p = 0.076. and overall classification accuracy = 63.0%.*

**Table 5:** Associations Between Alcohol Consumption Frequency and Sociodemographic Characteristics.

Variable	Category	No, *n* (%)	Daily, *n* (%)	2–3×/Week, *n* (%)	≥4×/Week, *n* (%)	≤1×/Month, *n* (%)	2–4×/Month, *n* (%)	χ^2^	*p*-Value	Effect Size
**Sex**	**Female**	597 (80.6%)	9 (1.2%)	26 (3.5%)	6 (0.8%)	66 (8.9%)	37 (5.0%)	43.400	<0.001	V = 0.181
	**Male**	382 (65.5%)	11 (1.9%)	47 (8.1%)	18 (3.1%)	82 (14.1%)	43 (7.4%)			
**Age group**	**≤25 years**	318 (70.0%)	4 (0.9%)	26 (5.7%)	8 (1.8%)	66 (14.5%)	32 (7.0%)	40.914	0.004	V = 0.096
	**26–30 years**	88 (68.2%)	3 (2.3%)	10 (7.8%)	3 (2.3%)	13 (10.1%)	12 (9.3%)			
	**31–39 years**	122 (73.5%)	6 (3.6%)	14 (8.4%)	2 (1.2%)	18 (10.8%)	4 (2.4%)			
	**40–49 years**	118 (76.6%)	3 (1.9%)	9 (5.8%)	1 (0.6%)	11 (7.1%)	12 (7.8%)			
	**≥50 years**	170 (85.0%)	3 (1.5%)	7 (3.5%)	1 (0.5%)	9 (4.5%)	10 (5.0%)			
**Occupation**	**Unemployed**	232 (88.2%)	4 (1.5%)	7 (2.7%)	2 (0.8%)	12 (4.6%)	6 (2.3%)	84.184	<0.001	V = 0.117
	**Student**	324 (70.4%)	3 (0.7%)	23 (5.0%)	8 (1.7%)	68 (14.8%)	34 (7.4%)			
	**Public-sector employee**	132 (66.3%)	2 (1.0%)	13 (6.5%)	9 (4.5%)	21 (10.6%)	22 (11.1%)			
	**Self-employed**	129 (63.2%)	6 (2.9%)	21 (10.3%)	2 (1.0%)	31 (15.2%)	15 (7.4%)			
	**Retired**	88 (83.0%)	3 (2.8%)	5 (4.7%)	1 (0.9%)	6 (5.7%)	3 (2.8%)			
	**Private sector**	6 (85.7%)	0 (0.0%)	0 (0.0%)	0 (0.0%)	1 (14.3%)	0 (0.0%)			
**Marital status**	**Married**	471 (81.6%)	10 (1.7%)	21 (3.6%)	7 (1.2%)	42 (7.3%)	26 (4.5%)	38.056	<0.001	V = 0.173
	**Single**	464 (67.1%)	10 (1.4%)	49 (7.1%)	14 (2.0%)	105 (15.2%)	50 (7.2%)			
**Education level**	**Primary/Secondary school**	163 (91.6%)	5 (2.8%)	4 (2.2%)	1 (0.6%)	3 (1.7%)	2 (1.1%)	91.307	<0.001	V = 0.133
	**High school**	255 (81.0%)	4 (1.3%)	16 (5.1%)	5 (1.6%)	22 (7.0%)	13 (4.1%)			
	**Undergraduate**	488 (69.0%)	7 (1.0%)	44 (6.2%)	13 (1.8%)	100 (14.1%)	55 (7.8%)			
	**Master’s**	42 (53.8%)	3 (3.8%)	4 (5.1%)	4 (5.1%)	19 (24.4%)	6 (7.7%)			
	**Doctorate**	11 (73.3%)	0 (0.0%)	3 (20.0%)	0 (0.0%)	0 (0.0%)	1 (6.7%)			
**Place of residence**	**Village**	29 (80.6%)	3 (8.3%)	2 (5.6%)	1 (2.8%)	0 (0.0%)	1 (2.8%)	45.856	<0.001	V = 0.110
	**Town**	18 (64.3%)	1 (3.6%)	6 (21.4%)	0 (0.0%)	0 (0.0%)	3 (10.7%)			
	**District**	142 (81.1%)	4 (2.3%)	6 (3.4%)	2 (1.1%)	11 (6.3%)	10 (5.7%)			
	**City**	751 (73.3%)	11 (1.1%)	55 (5.4%)	19 (1.9%)	129 (12.6%)	59 (5.8%)			
**Monthly income**	**<25,000 TRY**	204 (80.6%)	3 (1.2%)	7 (2.8%)	2 (0.8%)	22 (8.7%)	15 (5.9%)	29.092	0.016	V = 0.089
	**25,000–50,000 TRY**	152 (77.9%)	0 (0.0%)	11 (5.6%)	5 (2.6%)	19 (9.7%)	8 (4.1%)			
	**50,001–100,000 TRY**	150 (69.8%)	4 (1.9%)	10 (4.7%)	4 (1.9%)	36 (16.7%)	11 (5.1%)			
	**>100,000 TRY**	381 (69.4%)	10 (1.8%)	41 (7.5%)	11 (2.0%)	62 (11.3%)	44 (8.0%)			

*Note: Percentages are row percentages. V: Cramér’s V. Pearson chi-square p-values are reported.*

**Table 6:** Tobacco Product Use According to Sociodemographic Characteristics.

Variable	Category	Yes, *n* (%)	No, *n* (%)	χ^2^	*p*-Value	Effect Size
**Sex**	**Female**	171 (23.0%)	572 (77.0%)	97.217	<0.001	φ = 0.271
	**Male**	284 (49.0%)	296 (51.0%)			
**Age group**	**≤** **25 years**	132 (29.1%)	321 (70.9%)	20.401	<0.001	V = 0.136
	**26–30 years**	43 (33.6%)	85 (66.4%)			
	**31–39 years**	76 (45.2%)	92 (54.8%)			
	**40–49 years**	65 (42.5%)	88 (57.5%)			
	**≥** **50 years**	60 (30.2%)	139 (69.8%)			
**Occupation**	**Unemployed**	65 (24.6%)	199 (75.4%)	52.581	<0.001	V = 0.206
	**Student**	128 (27.8%)	332 (72.2%)			
	**Public employee**	80 (40.0%)	120 (60.0%)			
	**Self-employed**	104 (52.0%)	96 (48.0%)			
	**Retired**	41 (38.7%)	65 (61.3%)			
	**Private sector**	1 (14.3%)	6 (85.7%)			
**Marital status**	**Married**	200 (34.8%)	375 (65.2%)	0.173	0.678	φ = 0.012
	**Single**	233 (33.7%)	459 (66.3%)			
**Education level**	**Primary/Secondary school**	47 (26.4%)	131 (73.6%)	10.679	0.030	V = 0.091
	**High school**	127 (40.1%)	190 (59.9%)			
	**Undergraduate**	239 (34.0%)	464 (66.0%)			
	**Master’s**	24 (30.8%)	54 (69.2%)			
	**Doctorate**	4 (25.0%)	12 (75.0%)			
**Place of residence**	**Village**	15 (41.7%)	21 (58.3%)	2.169	0.538	V = 0.041
	**Town**	12 (42.9%)	16 (57.1%)			
	**District**	56 (32.0%)	119 (68.0%)			
	**City**	352 (34.4%)	670 (65.6%)			
**Monthly income**	**<25,000 TRY**	76 (29.9%)	178 (70.1%)	15.506	0.001	V = 0.113
	**25,000–50,000 TRY**	55 (28.2%)	140 (71.8%)			
	**50,001–100,000 TRY**	73 (34.0%)	142 (66.0%)			
	**>100,000 TRY**	224 (41.0%)	322 (59.0%)			

*Note: Percentages are row percentages. φ: Phi coefficient for 2 × 2 tables; V: Cramér’s V for larger contingency tables. Pearson chi-square p-values are reported.*

## Data Availability

The datasets generated and analyzed during the current study are available from the corresponding author upon reasonable request.
